# Interaction of Reactive Gases with Platinum Aerosol Particles at Room Temperature: Effects on Morphology and Surface Properties

**DOI:** 10.3390/nano11092266

**Published:** 2021-08-31

**Authors:** Vinzent Olszok, Malte Bierwirth, Alfred P. Weber

**Affiliations:** Institute of Particle Technology, Clausthal University of Technology, Leibnizstrasse 19, D-38678 Clausthal-Zellerfeld, Germany; vinzent.olszok@tu-clausthal.de (V.O.); malte.bierwirth@tu-clausthal.de (M.B.)

**Keywords:** platinum, spark discharge, morphology, sintering, Hydrogen, fractal dimension, aerosol photoemission spectroscopy, electron work function

## Abstract

Nanoparticles produced in technical aerosol processes exhibit often dendritic structures, composed of primary particles. Surprisingly, a small but consistent discrepancy was observed between the results of common aggregation models and in situ measurements of structural parameters, such as fractal dimension or mass-mobility exponent. A phenomenon which has received little attention so far is the interaction of agglomerates with admixed gases, which might be responsible for this discrepancy. In this work, we present an analytical series, which underlines the agglomerate morphology depending on the reducing or oxidizing nature of a carrier gas for platinum particles. When hydrogen is added to openly structured particles, as investigated by tandem differential mobility analysis (DMA) and transmission electron microscopy (TEM) analysis, Pt particles compact already at room temperature, resulting in an increased fractal dimension. Aerosol Photoemission Spectroscopy (APES) was also able to demonstrate the interaction of a gas with a nanoscaled platinum surface, resulting in a changed sintering behavior for reducing and oxidizing atmospheres in comparison to nitrogen. The main message of this work is about the structural change of particles exposed to a new environment after complete particle formation. We suspect significant implications for the interpretation of agglomerate formation, as many aerosol processes involve reactive gases or slightly contaminated gases in terms of trace amounts of unintended species.

## 1. Introduction

The formation of gas-borne aggregates from a cooling cloud of metal vapor has been investigated experimentally and in simulations in numerous studies [1,2,3,4,5]. For many common synthesis methods, the aggregates are composed of nanometer-sized primary particles and exhibit dendrite-like structures, which form under the action of diffusional aggregation. In this context, the fractal dimension (D_f_) of a particle is particularly well suited for studying gas-particle interactions, since various approaches are described to simulate fractal dimensions for different particle aggregation theories [6,7] and the simplicity of determination, e.g., for spark discharge generated particles [8]. D_f_ links directly to particle morphology since a low D_f_, i.e., close to 1.8, emphasizes openly structured particles and D_f_ = 3 or D_f_ = 2 corresponds to spheres in a mass-related analysis or projection area-related analysis, respectively. For mass mobility exponent determinations (same validity as D_f_), not fully explainable experimental results are reported with regard to particle aggregation theory showing an overestimation of mass mobility exponents for gold [9], suggesting a possible influence of the gaseous atmosphere, or trace components of the atmosphere, on particles during aggregation.

In detail, carrier gases in aerosol research typically exhibit technical purity or high purity with respect to gaseous contaminants. Regarding the purity of technical nitrogen or other carrier gases, the entire absence of oxygen can barely be reached. However, particle synthesis and analysis in ultrahigh vacuum (UHV) allows working under defined conditions with a low level of gaseous impurities [10]. Under the given circumstances, residual oxygen might be a disadvantage for the production of technical oxygen-free particles under atmospheric pressure. Especially for the preservation of an oxide-free metallic particle surface, hydrogen is a cheap and effective scavenger for free, adsorbed or chemically bonded oxygen. Without hydrogen, even nitrogen with a purity level of 6.0 (99.9999% and <0.5 ppm O_2_) forms oxides on a particle surface [11]. Thus, the use of hydrogen combines the advantages of low oxygen activity and a technically simple synthesis under atmospheric pressure conditions, avoiding UHV. Nevertheless, activation of hydrogen is always required for intercepting free oxygen, to reduce metal oxides on surfaces, as well as for capturing adsorbed oxygen through activated hydrogen species by means of a reduction of Gibbs free energy ΔG [12]. A thermal activation in a tube furnace might cause the same effect as an activation in a plasma by, e.g., dielectric barrier discharge [12,13,14]. It may be expected that an activation of hydrogen in the presence of particles always leads to morphological changes due to a thermal input. This leads to the open question about the role of reactive gases during the particle formation on aggregate morphology and particle surface properties. Moreover, it is unknown if the later addition of such gases can change the particle morphology even at room temperature. This question has so far not been addressed but is of significant importance for aerosols, i.e., particles dispersed in a carrier gas.

This paper focuses on the change in morphology and altered surface properties of platinum particles that were produced via spark discharge under 99.999% nitrogen. Platinum nanoparticles are known as highly active catalysts for different purposes and still attract interest within the aerosol community, even for the production of gas sensors [15]. Here, openly structured platinum agglomerates with a primary particle size of 4 nm were used for the investigation of gas adsorption behavior and sintering performance, especially for the interaction with hydrogen at room temperature. A tandem differential mobility analyzer setup was used to preselect a narrowly distributed fraction of the initial particle size distribution (PSD) to study the influences of different gases on the particle structure. Additionally, Aerosol Photoemission Spectroscopy (APES) was applied to determine the electron work function (eWF) of an aerosol, enabling an evaluation of surface changes as the measurement of eWF allows the assessment of solid–gas interactions as reported, among others, for reactive lithium surfaces [16].

## 2. Materials and Methods

The particle synthesis was performed by spark ablation in a so-called Spark Discharge Generator (SDG), consisting of a high voltage power supply, a charging capacitor (C = 24 nF) and two opposing metal electrodes. Between those platinum electrodes (purity 99.99%), a spark ablates a small amount of material, causing an oversaturated metal vapor to form primary particles while cooling down. Cooling rates in the order of ~10^8^ K/s lead to openly structured metal nanoparticles [17]. These openly structured particles can be described by Equation (1) with N the number of primary particles in one agglomerate, k as a prefactor, x_agglo_ as the agglomerate size, x_PP_ as the primary particle size and fractal dimension D_f_:

N = k (x_agglo_/x_PP_)^Df^ (1)

**Figure 1:**
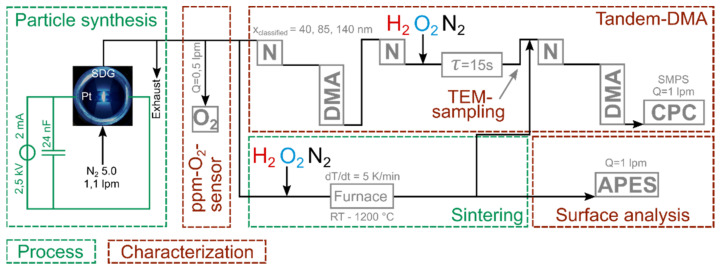
Experimental setup of particle synthesis, trace oxygen sensor, tandem DMA, sintering via tube furnace and APES. The admixture of reactive gases takes place seconds after the particle formation is completed, corresponding to the residence time of the aerosol between synthesis and point of gas addition. SDG: Spark discharge generator, O_2_: Trace oxygen sensor, DMA: Differential mobility analyzer, N: Soft X-ray neutralizer, CPC: Condensation particle counter, APES: Aerosol photoemission spectroscopy. The interconnection of N, DMA and CPC forms a Scanning Mobility Particle Sizer (SMPS).

**Figure 2:**
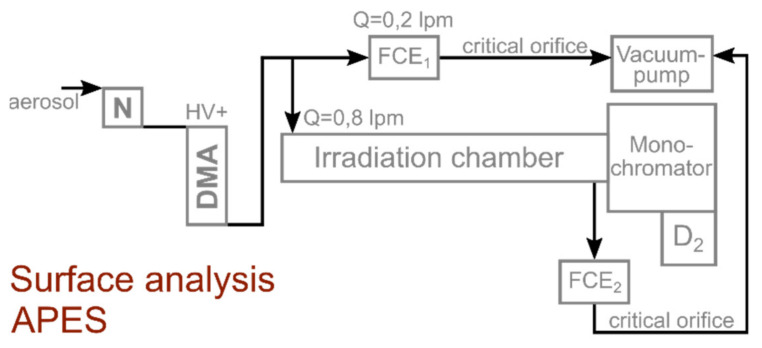
Detailed setup of APES. The spectral dispersion of the monochromatic light is 4.04 nm ± 0.13 nm from 190 nm to 320 nm. The standard deviation of eWF determination is in the range of ± 0.02 eV and is highly reproducible. N: Soft X-ray neutralizer, DMA: Differential mobility analyzer, FCE: Faraday cup electrometer, D_2_: Deuterium lamp.

The so-formed agglomerates are composed of aggregates of smaller size. In contrast to aggregates, which are distinguished by stronger bonds between primary particles, due to “necking” or partial particle melting, agglomerates can additionally include primary particles with weaker interparticle bonds. For all experiments, the carrier gas flow was kept constant at 1.1 lpm, as well as the breakthrough voltage at 2.5 kV, and the charging current at 2 mA. As depicted in Figure 1, the entire setup consists of five sections. The **particle synthesis** as described beforehand, a **trace oxygen sensor** (MESA GmbH, Filderstadt-Bonladen, Germany), the interconnection of two DMAs (model 3081, TSI Inc., Shoreview, Shoreview, MN, USA) including three soft X-ray Neutralizers (model 3088, N, TSI Inc., Shoreview, Shoreview, MN, USA) and a condensation particle counter (model 3010, CPC, TSI Inc., Shoreview, Shoreview, MN, USA) forming a **tandem DMA setup**, a **tube furnace sintering** (Nabertherm GmbH, Lilienthal, Germany, glass tube dimensions d = 26 mm, L = 70 cm) and an assembly for particle **surface analysis by APES**. Figure 2 shows a detailed view of the APES setup, including a broad-band UV-light source (deuterium lamp, D_2_, Bentham Instruments Ltd., Berkshire, UK), and a monochromator (Bentham Instruments Ltd., Berkshire, UK) that transmits well-defined monochromatic light into an irradiation chamber, where the charged aerosol interacts with photons of a certain energy. Since the particles were classified in a DMA before, they carry a single elementary charge (here chosen to be negative). When the photon energy equals or exceeds the eWF of the aerosol material, the singly charged particle emits an electron, with a certain probability (given by the photoelectric yield *Y*) and becomes undetectable by the second Faraday Cup Electrometer (FCE, SEADM S.L., Spain) downstream of the irradiation chamber. The photoelectric yield can be calculated by the Fowler-Nordheim equation (cf. Equation(2)) with c as the emission constant, hν as the photon energy and Φ as the eWF. Based on the concentration ratio of FCE_1_ and FCE_2_, the photoelectric yield *Y,* which is the probability of electron emission per photon incident on the particle, can be calculated. The plot of *Y^1/2^* versus the photon energy gives an S-shaped curve with a linear section that allows the extraction of the eWF at the intersection of the linear regression with the zero-Yield line [18]:

Y = c (hν − Φ)^2^ (2)

In order to run the SDG with a constant flow of nitrogen (Linde, 99.999%), the individual analytical sections were connected and disconnected for the intended measurements. As reactive gases, hydrogen was used as a premixed gas (Linde, 10% H_2_ & 90% N_2_) and oxygen as synthetic air (Linde, 20% O_2_ & 80% N_2_). The applied flow rate of hydrogen or oxygen was set constant at 0.2 lpm. If no reactive gas was added to the carrier gas, pure nitrogen was inserted instead of H_2_ or O_2_ mixtures at 0.2 lpm, respectively. The resulting H_2_ concentration in the process gas was about 2%, the O_2_ concentration about 4%. For the sake of simplicity, only the gas type is mentioned in the following figures, which refer to the abovementioned concentrations in the experiment.

For imaging of platinum nanoparticles, a Transmission Electron Microscope (TEM, Jeol, Tokyo, Japan) was used with finder-TEM grids (Plano) to deposit particles under nitrogen followed by taking of micrographs followed by a hydrogen gas treatment of the deposited particles and a last TEM analysis of exactly the same particles analyzed for nitrogen deposition. Thus, the finder-TEM grids allow superimposing one particle before and after a hydrogen treatment. Differences in the superimposed particle images indicate changes to the particles as a whole or parts of the particle, i.e., branches.

## 3. Results

The subsequent sections divide the result of the gas-particle interaction by discussing the thermal sintering behavior and the particle size measurements by tandem DMA, followed by TEM image analysis and particle surface investigations by APES.

### 3.1. Influence on Mobility Equivalent Particle Size

**Figure 3:**
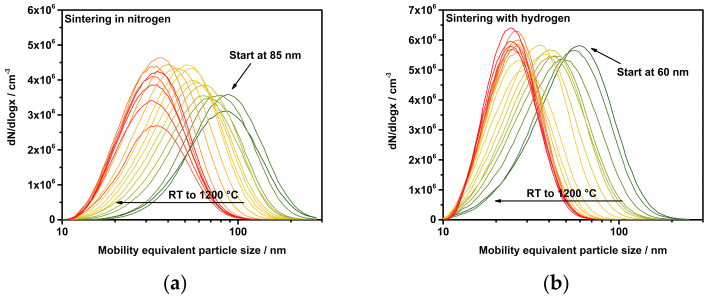
Sintering curves for platinum particles in nitrogen (**a**) and with hydrogen (**b**).

**Figure 4:**
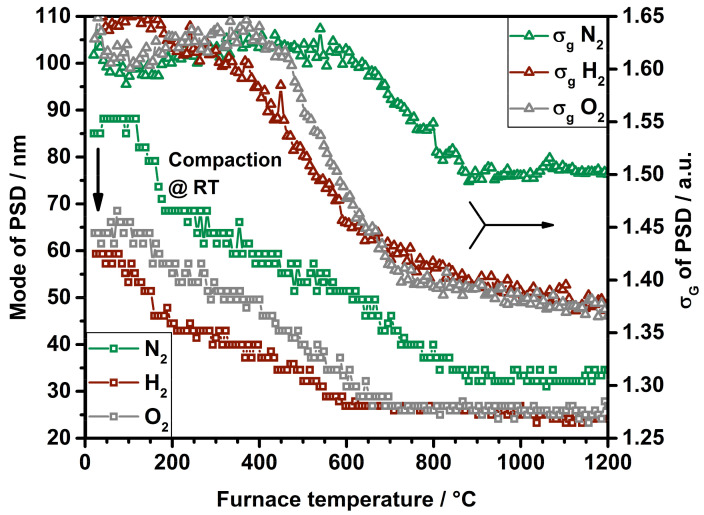
Mode and σ_g_ of PSDs for particle sintering in N_2_, with H_2_ and with O_2_

The first results on the sintering behavior of platinum particles under two different atmospheres are depicted in Figure 3. After admixing the reactive gases, the particles were introduced into the tube furnace, which was ramped up from room temperature (RT) to 1200 °C at 5 K/min. The subsequent Scanning Mobility Particle Sizer (SMPS) measured a particle size distribution every 90 s. It is important to emphasize here that the gases were added to the particles when their formation was completed, i.e., the primary particles had already aggregated and agglomerated. It is immediately apparent that already the first particle size distribution at room temperature with added hydrogen differs from pure nitrogen. The same behavior was observed with oxygenated process gas, which is included in Figure 4. For instance, the mode diameter decreased from 85 nm to 60 nm when hydrogen was added at room temperature. This decrease in size could not be reasoned by changes in the physical properties of the carrier gas such as different flow rates. For instance, the addition of hydrogen changed the flow rate through the CPC by 0.3% (0.991 lpm in N_2_ to 0.994 lpm with H_2_). Therefore, another phenomenon has to cause the size reduction.

Figure 4 displays the mode of the particle size distributions (left *y*-axis) and the related geometric widths σ_g_ of the distributions for the assumption of log-normal distributed agglomerate particles (right *y*-axis).

As stated before, the SDG produces particles under constant conditions. The initial particle size distribution exhibits a mode of about 85 nm that is reduced to 60 nm for H_2_ and 64 nm for O_2_. This leads to the assumption that adsorbing hydrogen as well as oxygen is able to densify the particle structure without an addition of thermal energy. In the course of the sintering curves for H_2_ and O_2_, it is visible that the sintering reaches a horizontal plateau at lower temperatures compared to the size decrease of platinum under nitrogen. A clear indicator of a modified sintering is given by the decrease in the width of the measured particle size distributions. The presence of a reactive gas leads to a narrower distribution, as displayed by the plots of σ_g_. Even though the particle synthesis is identical for all conditions, the particle sintering in presence of nitrogen ends up with a σ_g_ of 1.5. The curves for H_2_ and O_2_ fall even lower, to a geometric width of under 1.4, implying a similar sintering for both reactive gases. The gases are added to the particles long after the particle formation is completed. Therefore, the significant difference in σ_g_ between reactive and inert gases can be completely attributed to the surrounding gas atmosphere during sintering. Hydrogen and oxygen may improve the surface diffusivity of mobile platinum atoms during thermal restructuring. This could lead to more homogeneously formed particles in atmospheres containing reactive gases. A gas-dependent reorganization of platinum crystal facets was already reported by Altantzis et al., showing an increase in higher-order facets for oxygenated atmospheres, ending up with rounder particles. A hydrogen-containing environment led to lower-order faceted particles [19]. These investigations support the assumption that a gas can intervene in the restructuring process, as shown here by agglomerate sintering.

**Figure 5:**
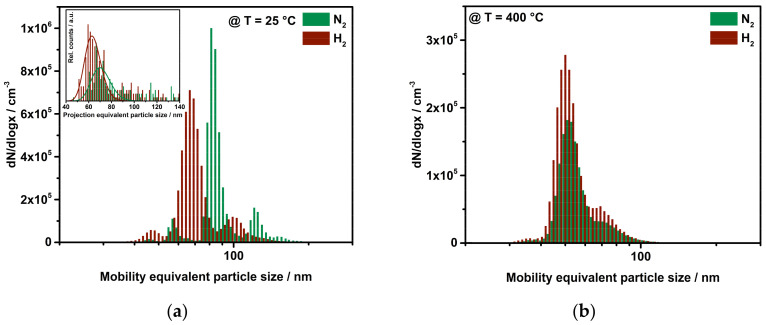
PSD obtained from tandem DMA. (**a**) A hydrogen gas treatment results in a shift to smaller agglomerates. The inset shows the projection equivalent diameter, acquired by TEM analysis, confirming a compaction. (**b**) Particles that underwent sintering at 400 °C are assumed to be mechanically reinforced and, therefore, unaffected by reshaping.

The analysis of the sintering behavior of platinum particles shows clearly an interaction between solid aerosol particles and the gaseous environment. Mutual movement of individual primary particles, folding of entire branches or fragmentation are possible hypotheses for the observed size reduction. Fragmentation would result in an increased number of smaller particles compared to the original PSD. An individual movement of primary particles as well as folding of branches would lead to a shift of the whole PSD towards smaller agglomerates. To better classify the possible mechanisms, tandem DMA experiments were performed. The original ensemble was classified to a monomobile fraction via a first DMA to 40 nm, 85 nm and 140 nm. After the classification, the charged aerosol was Boltzmann-neutralized by a second soft X-ray Neutralizer before a reactive gas was added. After a mixing and equilibrium phase in a glass tube (residence time of ca. 15 s), the PSD was measured via SMPS. The influence of hydrogen on the monomobile fraction of platinum particles is depicted in Figure 5a for RT and in Figure 5b for 400 °C. Additional SMPS scans confirm a consistent shift towards smaller agglomerate sizes for 40 nm and 140 nm classified particles at RT as well (not shown here). A gas-induced fragmentation of particles seems rather unlikely, since the shape of the particle size distribution and the total concentration remain constant.

The tandem DMA experiments were repeated for an oxygen-containing process gas as the sintering curves also showed a reduced particle size for RT in the presence of oxygen. Despite constantly and precisely running particle synthesis and monitoring of oxygen concentrations in the DMA sheath gas (about 8 ppm) and the whole system, it was not possible to verify a compaction of platinum particles in an O_2_-enriched atmosphere in the tandem DMA setup. On the one hand, it is presumed that reactive species emitted by the plasma between the SDG’s opposing metal electrodes adsorb on the particle surface. Those species might react with the added oxygen in the sintering setup (see Figure 1), resulting in a decreased size and compacted morphology. Unfortunately, using Fourier-Transform Infrared Spectroscopy (FTIR) and mass spectrometry (MS) with a resolution of around 1 ppm, no activated gas species could be detected. This may lead to two assumptions: either are not detectable due to low concentration or they are not present at all. On the other hand, residual oxygen in the sheath gas of the first DMA may be able to interact with the platinum particles in the moment when the particles enter the first DMA. A gas-induced compaction may already occur here, resulting in an unaffected particle size distribution when extra oxygen is added in the tandem DMA setup.

The fact that oxygen is adsorbed on the surface of the platinum particles can also be confirmed by O_2_-concentration measurements in the nitrogen entering and leaving the SDG. A trace oxygen sensor measures the concentration of the remaining oxygen in 99.999% purity N_2_ to about 1.3 ppm. When the SDG is turned on, the level of residual oxygen decreases immediately from 1.3 ppm to 0.25 ppm. Based on the measured difference in oxygen concentration and the total available surface of platinum (calculated from the primary particle size, the agglomerate particle size and the aerosol number concentration), the adsorbed oxygen forms roughly one monolayer covering the surface. This finding will be discussed and detailed later in the context of the APES assessment.

### 3.2. TEM Imaging of Gas-Treated Particles and the Assesment of the Fractal Dimension

**Figure 6:**
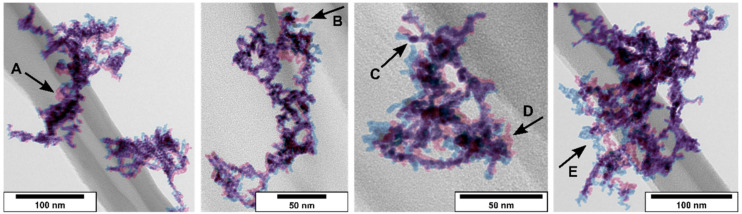
TEM micrographs for N_2_ (blue) and H_2_ (red). A, B, E: Shift of a compact part to another position. C: Rotation of a branch around a pivot point. D: Particle branch turns from the *z*-axis into the *x*-/*y*-axis of the micrograph.

As mentioned for the tandem DMA setup, compacting seems more likely for gas-treated particles than fragmentation. To confirm this hypothesis, TEM micrographs have been produced using finder grids. The following figure (Figure 6) shows four superimposed TEM micrographs. The blue-colored particles correspond to the particles produced under N_2_ in the SDG. The red-inked particles are the same particles shown in blue that underwent an H_2_ gas treatment by inserting the finder grid into the TEM sampling unit again with an applied gas flow of 0.2 lpm of 10% H_2_ in N_2_ for 15 s.

As marked by black arrows, a mobilization of certain branches could be observed. It is also noticeable that the morphology of the particles changes more significantly if a branch reaches out from the carbon fiber. Adhesive forces between particles and the fiber might pin certain parts of the particle. Free-hanging portions are more exposed to the by-flowing gas and are more mobile, resulting in an increase in compaction. Additionally, the movement of certain branches indicates loose contacts between two primary particles. For gold nanoparticles produced by spark discharge, so called “necking” can be seen due to ultra-pure and oxide free particles. A connection by means of a neck between two primary particles would lead to stable agglomerates that would not show any movement of branches, observed for presintered Pt particles (Figure 5b) and as reported for silver nanoparticles as well [20]. The observation of compaction in TEM analysis supports the idea of surface impurities due to residual oxygen in N_2_ 5.0 and PtO formation that prevents “necking”. Fragments, as well as particles close to the primary particle size, were not detected.

**Figure 7:**
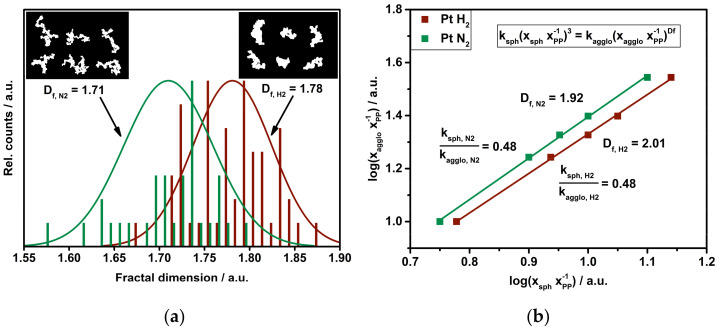
Fractal dimension of platinum particles under nitrogen and after exposure to hydrogen: (**a**) D_f_ estimation by TEM based on the determination of the radius of gyration. The insert shows binary TEM micrographs used for image analysis by *ImageJ*^®^. (**b**) Log-log plot of pre-classified platinum particles with an agglomerate size of x_agglo_ at room temperature, a primary particle size x_PP_ and spherically sintered particle size x_sph_ at 1150 °C. The slope of the linear fit equals 3/D_f_. Error bars are within the symbols.

Flow-through TEM grids also allow the sampling of platinum particles downstream of the mixing chamber for different added gases (see Figure 1). Figure 7a illustrates the change in morphology, expressed as an increase in D_f_ for particles freshly synthesized under N_2_ compared to particles exposed to H_2_. By comparison, the shift in D_f_ (ΔD_f,TEM_ = 0.07) fits perfectly with the results obtained from tandem DMA (ΔD_f,DMA_ = 0.09, Figure 7b), as both approaches show a compacting particle structure. The initial primary particle size (x_PP,N2_ = 4.1 nm and σ_g,N2_ = 1.09) for nitrogen equals, with respect to the resolution of the used TEM, the primary particle size for agglomerates treated with hydrogen (x_PP,H2_ = 3.9 nm and σ_g,H2_ = 1.09). Assuming the particle formation process to be a diffusion-limited cluster-cluster aggregation (DLCA) for spark discharge particle synthesis, Eggersdorfer and Pratsinis suggest a simulated D_f_ = 1.79 ± 0.03 for particles made of primary particles with σ_g_ = 1.00 [6]. This matches the measured fractal dimension for H_2_-treated Pt particles based on TEM analysis (see Figure 7). Finally, the change in D_f_ caused by admixed hydrogen shows a compacting particle structure, leading to the assumption that a gas is indeed capable of changing the morphological properties of agglomerates. Differences between estimated and experimentally demonstrated particle morphology, expressed by effective density or D_f_, might be strongly linked to gas–particle interaction after particle formation. Another indication can be found in the ratio of the prefactors k in the scaling laws for fractal-like aggregates (see Equation (1) and Figure 7b). Even though the fractal dimension increases with admixed hydrogen, the ratio of k_sph_/k_agglo_ remains constant. A reasonable explanation for this can be found in the previously discussed results. TEM analysis shows that certain branches of agglomerates are able to rotate around pivot points. Nevertheless, the stronger bonded aggregates within the agglomerate do not exhibit the same mobility. Therefore, the prefactor for the agglomerate is not influenced and the ratio stays the same, even with an increasing fractal dimension for admixed hydrogen.

An interim conclusion of the assessment of particle compaction results based on tandem DMA and TEM analysis leads to different possible approaches for an explanation of the interaction between platinum particles and reactive gases. It is reasonable to assume, on the one hand, that H_2_ that adsorbs on a platinum surface releases a certain amount of thermal energy corresponding to the adsorption process. This heat release might cause an increase in thermal mobility regarding adjacent primary particles, allowing them to move more easily against each other, resulting in a restructured morphology with a compacted appearance. As shown by trace oxygen measurements in the carrier gas leaving the SDG, a small amount of oxygen is preserved on the platinum particle surface. It is also likely, on the other hand, that a chemical reaction involving adsorbed oxygen and admixed H_2_ leads to water formation and a short-term weakening of primary particle/primary particle forces. The formed water would cover the surface of the particle homogenously, due to the water content of sub 2 ppm in the carrier gas immediate evaporation of produced water is likely. Torun et al. reported that even for hydrophilic SiO_2_ in low relative humidity, water coverage is no greater than one monolayer [21]. A possible monolayer of platinum oxide covering each primary particle might disappear when hydrogen is added, evoking the aforementioned increase in primary particle mobility during the fast hydrogen adsorption.

In order to support the proposed model of reshaping of platinum agglomerates due to gas interaction, a brief calculation of adsorption heat balanced to restructuring activation and van der Waals energies is intended. We assume two adjacent primary platinum particles with a diameter of 4 nm and a hydrogen adsorption heat release in the range of 96 kJ/mol for a surface coverage of 20% [22,23]. Based on the exposed platinum surface, a release of thermal energy of 4.8 × 10^−17^ J can be calculated. For oxygen, the adsorption heat is supposed to be even higher [24]. Furthermore, we assume a certain van der Waals (vdW) energy between two primary particles that are separated from each other by a distance of 4 Å, e.g., by the disappearance of platinum oxide when hydrogen is added. With a Hamaker coefficient of 2 × 10^−19^ J for platinum, an attractive vdW energy of 3.5 × 10^−20^ J results. It is already evident here that the thermal adsorption heat exceeds the vdW energy, supporting the model of an increased mobility leading to compaction. For Ag nanoparticle restructuring, Weber and Friedlander reported an activation energy of 2 × 10^−20^ J for 4 nm particles for temperature-induced compaction [25]. Based on the sintering behavior of Ni nanoparticles in hydrogen, Seipenbusch et al. stated an activation energy of 50 kJ/mol for an agglomerate to sinter, leading to a required energy of 5 × 10^−16^ J when we assume 40% of platinum atoms located on the particle surface of a 4 nm primary particle are involved in a restructuring process [26]. By comparison, the gas adsorption heat is higher than the activation energy suggested by Weber and Friedlander but lower than what is needed for the reshaping of Ni nanoparticles. Here, the difference between silver and platinum might be less than the difference between nickel compared to platinum. It is important to mention that Seipenbusch et al. studied the sintering of agglomerates into spheres, while this paper focuses on the compaction at room temperature. It seems likely that the interaction of gas molecules with a solid particle surface leads to a change in morphology just by adsorption and/or reaction processes evaluated based on energy considerations.

### 3.3. Surface Analysis by APES

**Figure 8:**
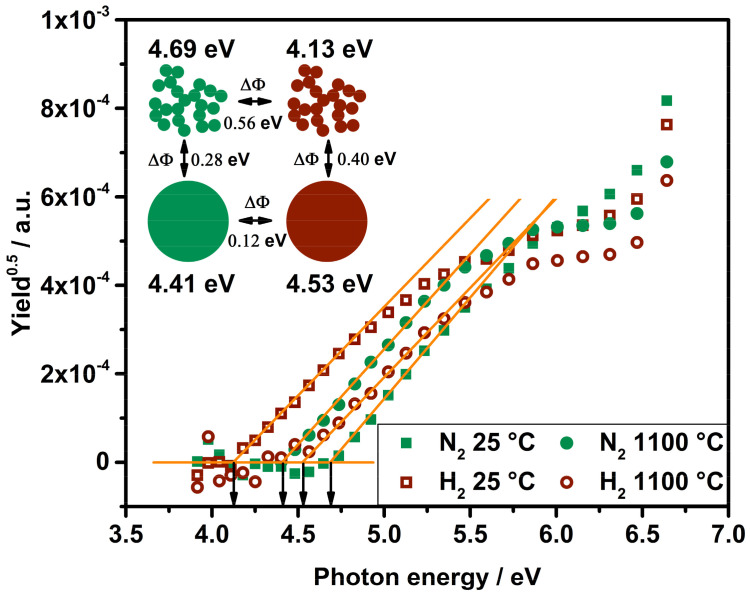
Work function determination of platinum particles in different atmospheres and with different morphologies.

In order to analyze the interaction between a particle and the surrounding gas, measurements of the eWF were conducted with APES. This online characterization technique takes advantage of the fact that the working principle of Aerosol Photoemission Spectroscopy is solely attributed to surface effects. An incident photon flux in combination with a residence time of five seconds within the irradiation chamber results in a certain yield for a chosen wavelength. The irradiance intensity-corrected and residence time-corrected *Y*^1/2^ are plotted in Figure 8 for platinum particles under room temperature and sintered spherical particles in N_2_ and with H_2_, respectively. The related eWF can be extracted at the intersection of the linear fit through the S-shaped curve and the zero-Yield line that represents an offset where particles do not change their charge state (orange horizontal line). Data points below the zero-yield are due to concentration fluctuations around the arithmetic average of particles, leaving the irradiation chamber in a charged state.

At room temperature, the agglomerate morphology has been found to change due to the presence of hydrogen. However, the primary particles appear unchanged in size and structure. That fact is indeed of significant relevance, as the eWF depends, among other aspects, on the curvature of an object. A decreasing eWF can be partly attributed to an increase in primary particle size [27]. For the performed APES measurements, it can be stated that a change in eWF results directly from a modification of the examined surface.

Comparing the respective measurements for nitrogen and hydrogen atmospheres, opposing trends become visible for the change in the eWF. As shown in Figure 8, platinum particles show a shift in eWF from 4.69 eV to 4.13 eV when hydrogen is attached to their surface at room temperature; thus, a reduction in eWF was measured. Apparently, hydrogen causes a decrease in eWF for room temperature particles, while the sintered platinum spheres exhibit an increase in eWF shifting from 4.41 eV to 4.53 eV. It has already been reported that adsorbed hydrogen can cause a decrease in eWF [28,29] but also an increase [29] depending on surface contamination. A sintering process can be seen as a change even for surface contamination, thus opposing trends do not appear contrary to the measured eWFs by APES.

Despite the reversed trend of eWFs for spheres synthesized in the presence of hydrogen, the shift of ΔΦ = 0.40 eV is even more significant than for nitrogen-containing atmosphere (ΔΦ = 0.28 eV). Trace oxygen measurements indicate that oxygen bonds to the particle surface; hence, the eWF of platinum particles under “pure” nitrogen equals a value corresponding to an oxide-covered particle. It is tempting to claim that the eWF measured under N_2_ 5.0 represents a clean and pure platinum surface, but the reported results lead to a different assessment. For perfectly clean bulk platinum single crystals measured in UHV, eWFs in the range from 5.6–6.4 eV are reported [30]. However, the APES results obtained here are intended to show differences only with regard to measurements under atmospheric pressure conditions. Taking the findings of Altantzis et al. into account, different gases support the formation of different crystal facets within a sintered particle [19]. The ability of removing an electron from a solid surface, described by the eWF, also depends on the crystal side where the electron detaches from resulting in different eWF for different crystal facets [30]. This fact might influence the evaluation and comparison of differences in eWF for different morphologies and temperature history.

Finally, the results obtained from APES should support two main statements: an interaction of hydrogen or oxygen with a nanoscaled platinum surface was observed by means of a change in eWF. A difference of ΔΦ = 0.56 eV for room temperature particles shows that hydrogen is able to be adsorbed on the particle surface. Furthermore, a difference of ΔΦ = 0.12 eV for sintered particles shows the oxygen to be part of the investigated system as platinum oxide formation seems realistic based on trace oxygen measurements, but the behavior of thermally instable PtO needs to be discussed and evaluated with respect to APES in the future.

## 4. Conclusions

Initial investigations on the sintering behavior of fractal-like platinum particles under nitrogen, added hydrogen and with oxygen yielded unexplained results, as the first particle size distribution displayed a reduced agglomerate size for particles that were in contact with hydrogen or oxygen at room temperature, respectively. A tandem DMA setup offered the chance to study the restructuring of particles when reactive gases were added seconds after complete particle formation. The fractal dimension was found to increase as the agglomerate size decreased due to the influence of hydrogen, also observed with TEM. Two possible explanations were given for the movement of primary particles. The adsorption heat of hydrogen on platinum might deliver enough thermal energy to overcome the van der Waals energy between two primary particles, ending up with a new position of a branch. The removal of small amounts of surface bonded oxygen by admixed hydrogen, and hence, the formation of water might cause a short-term weakening of the contact between the primary particles. Unfortunately, it was not possible to get insight into the oxygen-induced reshaping that was observed in the sintering setup only. This might be due to the need for ultra-pure and oxygen-free nitrogen in particle synthesis. It is assumed that activated species formed by the spark in the SDG play an important role in the oxygen-made compaction.

Nevertheless, important findings were obtained, as the interaction effect of freshly prepared particles with a gaseous atmosphere was demonstrated. In the future, more attention will have to be given to fractal-like particles when reducing or oxidizing gases are added to an aerosol. Especially for metallic nanoparticles, an influence on morphological as well as surface-related properties is expected, even for metals beyond platinum. It is hypothesized that in previous publications, a mismatch between theory and experiment might be due to the gas-particle interaction. Future investigation will focus on the online characterization of change in the fractal dimension by using more defined carrier gas compositions and by employing the system DMA/ICP-MS to get a deeper insight into agglomerate restructuring.

## Data Availability

The data is available on reasonable request from the corresponding author.

## Abbreviations

APES	Aerosol Photoemission Spectroscopy
TEM	Transmission Electron Microscopy
SMPS	Scanning Mobility Particle Sizer
DMA	Differential Mobility Analyzer;
SDG	Spark Discharge Generator
PSD	Particle size distribution
N	Soft X-ray neutralizer
RT	Room temperature
eWF	Electron Work Function
lpm	liters per minute
FTIR	Fourier-Transform Infrared Spectroscopy;
MS	Mass spectrometry
ICP-MS	Inductively Coupled Plasma Mass Spectrometry
UHV	Ultra High Vacuum
**Symbols**	
Q	Gas flow rate
x	Particle size
D_f_	Fractal dimension
Y	Yield
C	Capacitance
c	Emission constant
hν	Photon energy
ΔG	Gibbs free energy
Φ	Electron Work Function
k	Prefactor
